# Investigating the mechanism of impact and differential effect of the Quality Premium scheme on antibiotic prescribing in England: a longitudinal study

**DOI:** 10.3399/bjgpopen20X101052

**Published:** 2020-07-15

**Authors:** Philip Emeka Anyanwu, Koen Pouwels, Anne Walker, Michael Moore, Azeem Majeed, Benedict W J Hayhoe, Sarah Tonkin-Crine, Aleksandra Borek, Susan Hopkins, Monsey Mcleod, Céire Costelloe

**Affiliations:** 1 Department of Primary Care and Public Health, Imperial College London, London, UK; 2 Nuffield Department of Population Health, Health Economics Research Centre, University of Oxford, Oxford, UK; 3 Nuffield Department of Medicine, University of Oxford, Oxford, UK; 4 National Institute for Health Research (NIHR) Health Protection Research Unit in Healthcare Associated Infections and Antimicrobial Resistance, University of Oxford, Oxford, UK; 5 Primary Care, Population Sciences and Medical Education, University of Southampton, Southampton, UK; 6 Primary Care and Public Health, Imperial College London School of Public Health, London, UK; 7 Nuffield Department of Primary Care Health Sciences, University of Oxford, Oxford, UK; 8 Healthcare-Associated Infection and Antimicrobial Resistance Department, National Infection Service, Public Health England, London, UK; 9 Directorate of Infection, Royal Free London NHS Foundation Trust, London, UK; 10 Department of Infectious Disease, Imperial College London, London, UK; 11 Pharmacy Department, Centre for Medication Safety and Service Quality, Imperial College London, London, UK; 12 NIHR Patient Safety Translational Research Centre, Imperial College London, London, UK; 13 NIHR Health Protection Research Unit in Healthcare-Associated Infections and Antimicrobial Resistance, Imperial College London, London, UK

**Keywords:** anti-bacterial agents, quality premium, resistance, financial incentive, primary health care, general practice

## Abstract

**Background:**

In 2017, approximately 73% of antibiotics in England were prescribed from primary care practices. It has been estimated that 9%–23% of antibiotic prescriptions between 2013 and 2015 were inappropriate. Reducing antibiotic prescribing in primary care was included as one of the national priorities in a financial incentive scheme in 2015–2016.

**Aim:**

To investigate whether the effects of the Quality Premium (QP), which provided performance-related financial incentives to clinical commissioning groups (CCGs), could be explained by practice characteristics that contribute to variations in antibiotic prescribing.

**Design & setting:**

Longitudinal monthly prescribing data were analysed for 6251 primary care practices in England from April 2014 to March 2016.

**Method:**

Linear generalised estimating equations models were fitted, examining the effect of the 2015–2016 QP on the number of antibiotic items per specific therapeutic group age–sex related prescribing unit (STAR-PU) prescribed, adjusting for seasonality and months since implementation. Consistency of effects after further adjustment for variations in practice characteristics were also examined, including practice workforce, comorbidities prevalence, prescribing rates of non-antibiotic drugs, and deprivation.

**Results:**

Antibiotics prescribed in primary care practices in England reduced by -0.172 items per STAR-PU (95% confidence interval [CI] = -0.180 to -0.171) after 2015–2016 QP implementation, with slight increases in the months following April 2015 (+0.014 items per STAR-PU; 95% CI = +0.013 to +0.014). Adjusting the model for practice characteristics, the immediate and month-on-month effects following implementation remained consistent, with slight attenuation in immediate reduction from -0.172 to -0.166 items per STAR-PU. In subgroup analysis, the QP effect was significantly greater among the top 20% prescribing practices (interaction *p*<0.001). Practices with low workforce and those with higher diabetes prevalence had greater reductions in prescribing following 2015–2016 QP compared with other practices (interaction *p*<0.001).

**Conclusion:**

In high-prescribing practices, those with low workforce and high diabetes prevalence had more reduction following the QP compared with other practices, highlighting the need for targeted support of these practices and appropriate resourcing of primary care.

## How this fits in

The QP has previously been associated with reductions in antibiotic prescribing in primary care practices in England. To the authors’ knowledge, this study is the first to investigate the other possible explanations of the effect of the QP on antibiotic prescribing in primary care practices, strengthening the evidence on the effectiveness of this financial incentive scheme. The results show a consistent effect of QP after accounting for differences in practice characteristics, indicating its inclusiveness in reaching diverse populations. The study provides novel evidence on the differential effect of QP, emphasising the role of CCGs in identifying and supporting higher-prescribing practices, understaffed practices, and those dealing with a high prevalence of comorbidities.

## Introduction

The overuse of antibiotics drives resistance through the selection of antibiotic-resistant strains of organisms.^1–3^ Primary care is the main contributor to antibiotic usage in England, constituting approximately 73% of antibiotics prescribed in 2017.^4^ It is estimated that 9%–23% of antibiotic prescriptions in primary care practices in England between 2013 and 2015 were inappropriate, which is based on prescribing guidelines.^5^

Antibiotic prescribing is recommended in the management of respiratory tract infections (RTIs) in some patients, including older patients with diabetes or heart failure, who are considered at particular risk of developing complications.^6^ The prevalence of comorbidities such as diabetes varies over time and geographical area, contributing to the disparities in the antibiotic prescribing pattern within and between primary care practices.^7,8^

Several approaches have been adopted to reduce primary care antibiotic prescribing in England including: increased surveillance and prescribing feedback; the provision of C-reactive protein point-of-care testing; education and training interventions targeted at prescribers and patients; public antimicrobial stewardship (AMS) campaigns; and financial incentives.^9,10^ The QP is an NHS England performance-related incentive scheme established in 2013 to reward CCGs financially, based on the quality of specific health services considered to be national or local priorities and commissioned over a specific period.^9^ Individual GP practices did not receive financial remuneration in relation to this award. Reducing antibiotic prescribing in primary care was included as one of the national priorities in the 2015–2016 QP guidance, targeting a reduction of 1% of the mean value in England in 2013–2014 (1.161 antibiotic items per STAR-PU) for a CCG to obtain the financial incentive (provided other non-antibiotic targets, such as early cancer diagnosis, improving GP access and experience, continuing health care, and mental health, were also met).

The QP intervention has been associated with substantial reductions in antibiotic prescribing in primary care practices in England,^4,11–13^ specifically a reduction of about 2.7 million antibiotic items between 2014–2015 and 2016–2017 financial years (1 April to 31 March).^4^ Such reductions in antibiotic prescribing would be expected to contribute to reductions in the development of resistance.^14^

However, it is not clear whether these reported reductions can be entirely attributed to the QP as current evaluations have not accounted for other possible explanations, such as variations in practice characteristics over time. The main aim of this study was, therefore, to investigate whether differences in primary care practice characteristics, which can contribute to variance in antibiotic prescribing (practice workforce, the prevalence of comorbidities, prescribing rate on non-antibiotic drugs, and deprivation index), explained any of the effects of the QP scheme on the prescribing rates in primary care practices. Furthermore, the study investigated whether the QP had a differential effect on high-prescribing practices and other subgroups of practices.

## Method

### Study design

New QP guidance was implemented at the start of each financial year in England with changes in the targeted reductions in antibiotic prescribing and its associated award. This study adopted a natural experiment approach in investigating the mechanism of impact of the 2015–2016 QP target on antibiotic prescribing in primary care practices in England, with the preceding financial year as the control, by conducting analyses of longitudinal (monthly) prescribing data for 6251 primary care practices in England from April 2014 to March 2016. While there are more recent antibiotic prescribing data, including data beyond March 2016would have meant exceeding the period covered by the 2015–2016 QP guidance, as prescribing in subsequent periods are covered by different QP guidance and targets.

Like antibiotics, the prescription of opioids and benzodiazepines is monitored in the UK with prescribers encouraged to reduce their prescription.^15^ The frequency of prescribing of these drugs has been reported as an indicator of antibiotic prescribing rates.^16^ Adjusting for the prescribing behaviour of opioids and benzodiazepines was important to account for the overall medicine prescribing behaviour of practices that might not be specific to antibiotics.

### Variables

#### Outcome

The primary outcome was a continuous variable indicating the number of antibiotic items per STAR-PU prescribed by a practice in England per month. Practice-level antibiotic prescribing data were sourced from OpenPrescribing (an Evidence-Based Medicine DataLab project at the University of Oxford) and STAR-PU weighted using figures from the 2013 item-based age–sex weighting for oral antibacterials,^17^ and the number of registered patients in each age–sex category in a practice for each specific month.^18^

#### Predictors

The main predictor was a binary variable indicating the implementation of the 2015–2016 QP. The intervention period included April 2015 to March 2016 with the control period as the prior 12 months (that is, April 2014 to March 2015). A continuous variable representing the number of months since 2015–2016 QP implementation was used to examine changes in trend in the months following the intervention.

#### Confounder and effect modifiers

Confounder and effect modifiers in this study included:

- the number of GPs per 10 000 patients in each practice for each financial year (sourced from the NHS workforce data);^19^
- the Index of Multiple Deprivation (IMD), computed based on the lower super output area for each practice's postcode^20,21^ (this only accounts for the site of practice and not patient-level data);
- yearly prevalence of specific comorbidities per 100 patients, namely asthma, chronic obstructive pulmonary disease (COPD), diabetes mellitus (for type 1 and 2), cancer, and chronic kidney disease, from the NHS Quality and Outcomes Framework (QOF) database (QOF data do not distinguish between type 1 and type 2 diabetes mellitus).^22^ These comorbidities have been chosen as their prevalence is relevant to the antibiotic prescribing rate of a practice given that antibiotic prescribing is recommended in the management of some patients with RTI with these comorbidities;^6,23–25^ and,
- the monthly prescribing rate of opioids and benzodiazepines (per 100 patients) from practice level prescribing data published by NHS Digital.^26^

Data from the different sources were linked using practice code, which is a unique identifier for practices in England. Data from OpenPrescribing and NHS Digital are from the same source: NHS Business Services Authority prescribing and dispensing information systems.

As the analysis is at a monthly level, all models were adjusted for seasonality (using a categorical term with winter as the reference category) to account for the seasonal differences in antibiotic prescribing, with increased incidence of RTIs during the winter months associated with higher antibiotic prescribing.^27^

The dataset covered 7549 practices existing over the study period. 876 practices were dropped due to incomplete observation for all variables (11.6%; mostly practices that closed or opened during the study period) and then 422 practices (5.6%) that were outliers were dropped using the interquartile range rule; therefore, the final analyses included 6251 (82.8%) practices.

### Statistical analysis

Generalised estimating equations models were fitted with an autoregressive ‘AR(1)’ covariance structure^28^ to investigate the effect of the 2015–2016 QP on antibiotic prescribing. The first model included variables reflecting the 2015–2016 QP and the number of months since its implementation as the predictors, adjusting for seasonality.

To examine whether this estimated effect of the 2015–2016 QP on antibiotic prescribing was explained by differences in practice characteristics that can contribute to variance in antibiotic prescribing, variables were intoduced reflecting practice characteristics (workforce size, prevalence of comorbidities, rate of prescribing of non-antibiotic drugs, and IMD) to investigate whether the effect of the QP was retained, declined, or intensified.

In the multivariable model, variables causing multicollinearity were excluded (defined by opposite effects in univariable and multivariable models and high Spearman correlation >0.5). To address non-linearity, the association was modelled between the outcome and workforce using linear spline terms with knots equally spaced over the range of the workforce data (at 4.91, 9.81, and 14.72). A principal component analysis (PCA) was used to compute a summary score reflecting respiratory diseases comorbidity (using asthma and COPD prevalence). The PCA produced two components with eigenvalues of 1.41 and 0.59. The first component was retained (with an eigenvalue >1), which explained 71% of the variation in the data on respiratory diseases prevalence.

Subgroup analyses were also conducted using interaction terms to examine whether the 2015–2016 QP had a differential effect on antibiotic prescribing among: high-prescribing practices (the top 20% prescribing practices based on the mean antibiotic items per STAR-PU prescribed in 2014–2015); practices with a higher prevalence of comorbidities; low workforce; and in deprived areas. For more detailed analysis of the differential effect of QP based on levels of comorbidity prevalence, the study used linear spline functions with knots equally spaced over the range of the variables on diabetes prevalence (knots at 3.93, 7.60, and 11.28) and the PCA summary score for respiratory diseases (knots at -2.05, 0.32, and 2.70).

All analyses were conducted using Stata (version 15.1). Results for all models are presented as coefficients with 95% CIs.

## Results

The data constituted 150 024 observations for 6251 practices; each practice contributed data for 24 months. The mean number of antibiotic items prescribed in 2014–2015 QP was 1.106 items per STAR-PU (95% CI = 1.103 to 1.108); this was 0.097 items higher than the mean in the post-intervention period (Table 1). The top 20% prescribing practices had fewer GPs per 10 000 patients, higher rates of prescriptions for the non-antibiotic drugs, and higher prevalence of comorbidities (apart from cancer) compared with the entire population.

**Table 1.** Characteristics of general practices, *N* = 6251

	**Mean, entire study population,**2014–2015 & 2015–2016,% (95% CI)	**Mean, period before QP,**2014–2015,% (95% CI)	**Mean, period after QP,**2015–2016,% (95% CI)	**Mean, top 20% of prescribing practices,**2014–2015 & 2015–2016,% (95% CI)
Antibiotic items per STAR-PU	1.057(1.055 to 1.059)	1.106(1.103 to 1.108)	1.009(1.007 to 1.011)	1.357(1.354 to 1.360)
Asthma prevalenceper 100 patients	5.941(5.935 to 5.947)	5.980(5.971 to 5.989)	5.901(5.893 to 5.911)	6.295(6.281 to 6.309)
COPD prevalenceper 100 patients	1.878(1.873 to 1.882)	1.861(1.855 to 1.867)	1.894(1.888 to 1.900)	2.301(2.291 to 2.312)
Cancer prevalenceper 100 patients	2.346(2.342 to 2.350)	2.264(2.258 to 2.270)	2.428(2.421 to 2.434)	2.230(2.291 to 2.309)
CKD prevalenceper 100 patients	4.129(4.119 to 4.139)	4.143(4.129 to 4.157)	4.115(4.101 to 4.129)	4.420(4.398 to 4.442)
Diabetes prevalenceper 100 patients	6.635(6.626 to 6.645)	6.544(6.532 to 6.557)	6.726(6.713 to 6.740)	7.316(7.298 to 7.335)
Opioids prescriptionper 100 patients	3.273(3.265 to 3.282)	3.241(3.229 to 3.253)	3.306(3.293 to 3.318)	4.306(4.285 to 4.328)
Benzodiazepineanxiolytics prescriptionper 100 patients	0.917(0.914 to 0.919)	0.920(0.916 to 0.924)	0.913(0.910 to 0.917)	1.151(1.145 to 1.158)
Benzodiazepinehypnotics prescriptionper 100 patients	1.311(1.308 to 1.315)	1.333(1.328 to 1.338)	1.289(1.284 to 1.294)	1.673(1.664 to 1.682)
GP workforceper 10 000 patients	6.126(6.112 to 6.139)	6.429(6.412 to 6.446)	5.822(5.802 to 5.842)	5.835(5.805 to 5.864)

CKD = chronic kidney disease. COPD = chronc obstructive pulmonary disease. QP = Quality Premium. STAR-PU = specific therapeutic group age–sex related prescribing unit.

Univariable models showed the predictors and covariates (apart from the last spline term for workforce) were all significantly associated with the antibiotic prescribing rate in primary care practices in England (see Supplementary Table S1).

### Effect of the 2015–2016 QP on antibiotic prescribing (adjusting for seasonality)

**Figure 1.**
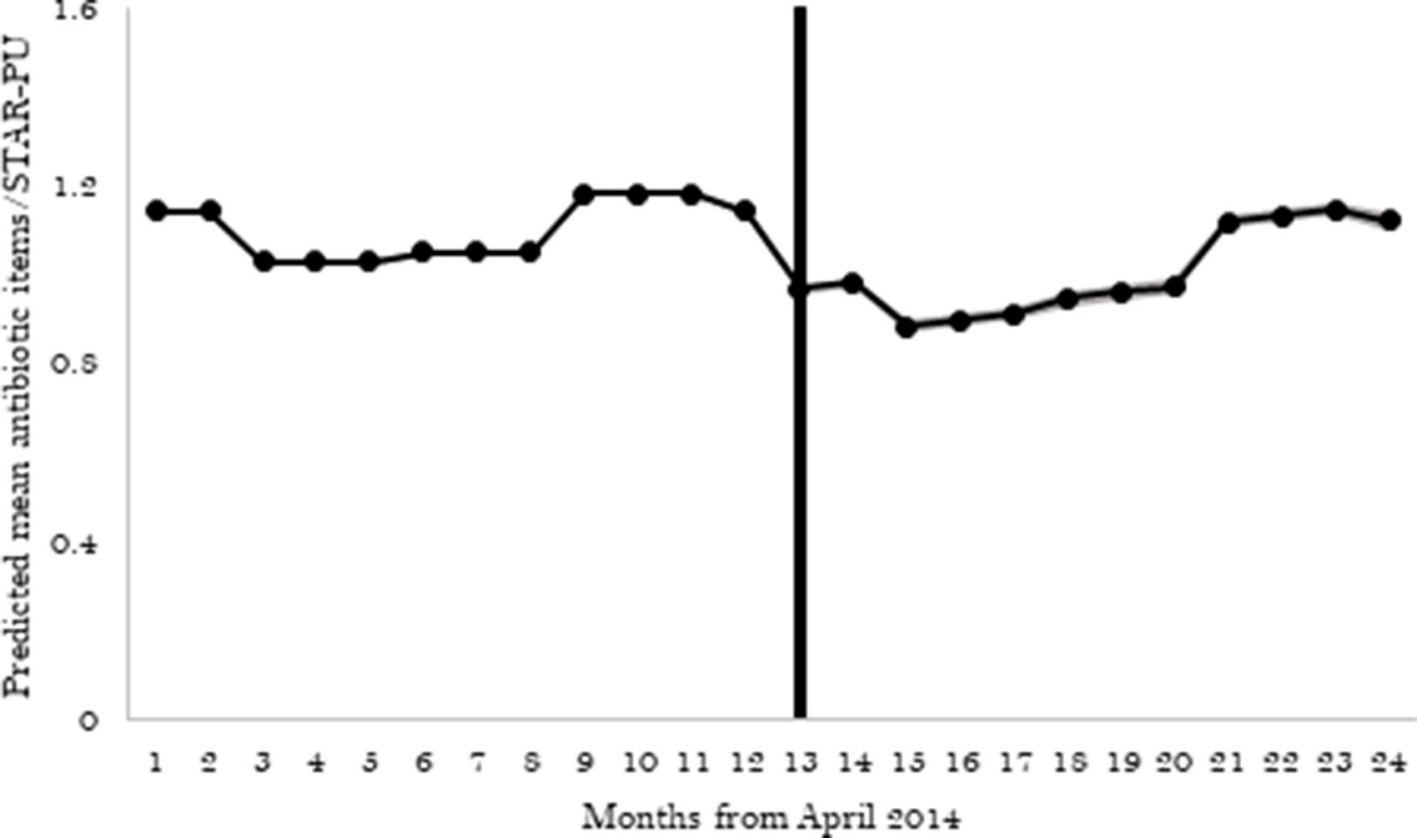
Effect of QP on antibiotic prescribing. The shaded portion around the line represents the 95% CI. The vertical line indicates implementation of the 2015–2016 QP in April 2015. STAR-PU = specific therapeutic group age–sex related prescribing unit

Antibiotic prescribing in general practices in England reduced by -0.172 items per STAR-PU (95% CI = -0.176 to -0.168) after 2015–2016 QP implementation in April 2015 compared with the 12 months before (Table 2 and Figure 1). There was a slight increase in the months following April 2015 (month-on-month increase +0.014 items per STAR-PU; 95% CI = +0.013 to +0.014).

**Table 2.** Association between QP and antibiotic prescribing, *N* = 6251

	**Model without adjustment for practice characteristics**	**Model with adjustment for** **practice characteristics**
**Co-efficient**	**95%** CI	**Co-efficient**	**95%** CI
**Lower**	**Upper**	**Lower**	**Upper**
2015–2016 QP		-0.172	-0.176	-0.168	-0.166	-0.170	-0.162
Months since QP		0.014	0.013	0.014	0.014	0.013	0.014
Season	Winter	Ref	Ref	Ref	Ref	Ref	Ref
	Spring	-0.040	-0.043	-0.038	-0.044	-0.046	-0.041
	Summer	-0.153	-0.156	-0.150	-0.139	-0.142	-0.136
	Autumn	-0.132	-0.135	-0.129	-0.119	-0.121	-0.116
Comorbidities	Respiratory disease	–	–	–	0.021	0.018	0.024
	Diabetes prevalence	–	–	–	0.028	0.026	0.030
Benzodiazepine anxiolytics prescription		–	–	–	0.123	0.118	0.127
Benzodiazepine hypnotics prescription		–	–	–	0.160	0.157	0.164
GPHC per 10 000 patients(spline terms)	GPHC1 (<4.91)	–	–	–	0.013	0.011	0.015
	GPHC2 (≥4.91 to ≤9.80)	–	–	–	-0.008	-0.010	-0.006
	GPHC3 (≥9.81 to ≤14.72)	–	–	–	0.07	0.002	0.011
	GPHC4 (>14.72)	–	–	–	-0.006	-0.021	0.008

Effects of GPHC are per one increase in GP number per 10 000 patients within each spline term. GPHC = GP headcount. QP = Quality Premium.

### Effect of 2015–2016 QP on antibiotic prescribing (further adjusting for practice characteristics)

After extending the model to adjust for practice characteristics (comorbidities [respiratory diseases and diabetes], prescribing rate of benzodiazepine, and GP workforce), the immediate and month-on-month effects seen after 2015–2016 QP implementation remained consistent, with only a slight attenuation in mean reduction in items prescribed immediately following the QP from -0.172 to -0.166 items per STAR-PU, a 3.5% relative reduction in effect size (Table 2).

### Subgroup analysis

With the subgroup analyses, the study examined whether the effect of the QP on antibiotic prescribing was greater within specific subgroups of practices, such as those that are among the top 20% prescribers, with a more complex patient population in relation to the prevalence of comorbidities, size of workforce, and deprivation index using interaction terms.

The study found a differential effect of the QP among the top 20% prescribers in 2014–2015. The reduction in antibiotic prescribing following the QP implementation was greater among the top 20% prescribers (-0.200 items per STAR-PU; 35.2% reduction from the rate before QP) compared with other practices (-0.116 items per STAR-PU; interaction *p*<0.001) (Table 3).

**Table 3.** Subgroup analysis

	**Co-efficient**	**95%** CI
**Lower**	**Upper**
2015–2016 QP in bottom 80% of prescribers		-0.116	-0.125	-0.106
Effect of 2015–2016 QP in top 20% of prescribers		-0.200	-0.210	-0.187
Top 20% of prescribers		0.309	0.302	0.316
Months since QP		0.013	0.013	0.014
Season	Winter	Ref	Ref	Ref
	Spring	-0.046	-0.049	-0.044
	Summer	-0.148	-0.151	-0.145
	Autumn	-0.124	-0.126	-0.121
Comorbidities prevalence per 100 patients	Respiratory diseases	0.013	0.011	0.015
	Diabetes (<3.93%)	0.097	0.089	0.106
	Diabetes (≥3.93% to ≤7.59%)	0.015	0.012	0.017
	Diabetes (≥7.60% to ≤11.28%) before QP	0.016	0.012	0.021
	Diabetes (≥7.60% to ≤11.28%) after QP	0.007	0.002	0.011
	Diabetes (>11.28%)	0.041	0.030	0.052
GPHC per 10 000 patients, spline terms	GPHC1 (<4.91) before QP	0.015	0.013	0.017
	GPHC1 (<4.91) after QP	0.007	0.005	0.009
	GPHC2 (≥4.91 to ≤9.80) before QP	-0.007	-0.009	-0.005
	GPHC2 (≥4.91 to ≤9.80) after QP	-0.005	-0.007	-0.003
	GPHC3 (≥9.81 to ≤14.72)	0.005	-0.000	0.010
	GPHC4 (>14.72)	-0.007	-0.022	0.008
Benzodiazepine anxiolytics prescription		0.093	0.089	0.097
Benzodiazepine hypnotics prescription		0.118	0.115	0.121

Effects of diabetes are per 1% higher within each spline term. Effects of GPHC are per 1 higher per 10 000 patients within each spline term. GPHC = GP headcount. QP = Quality Premium.

**Figure 2.**
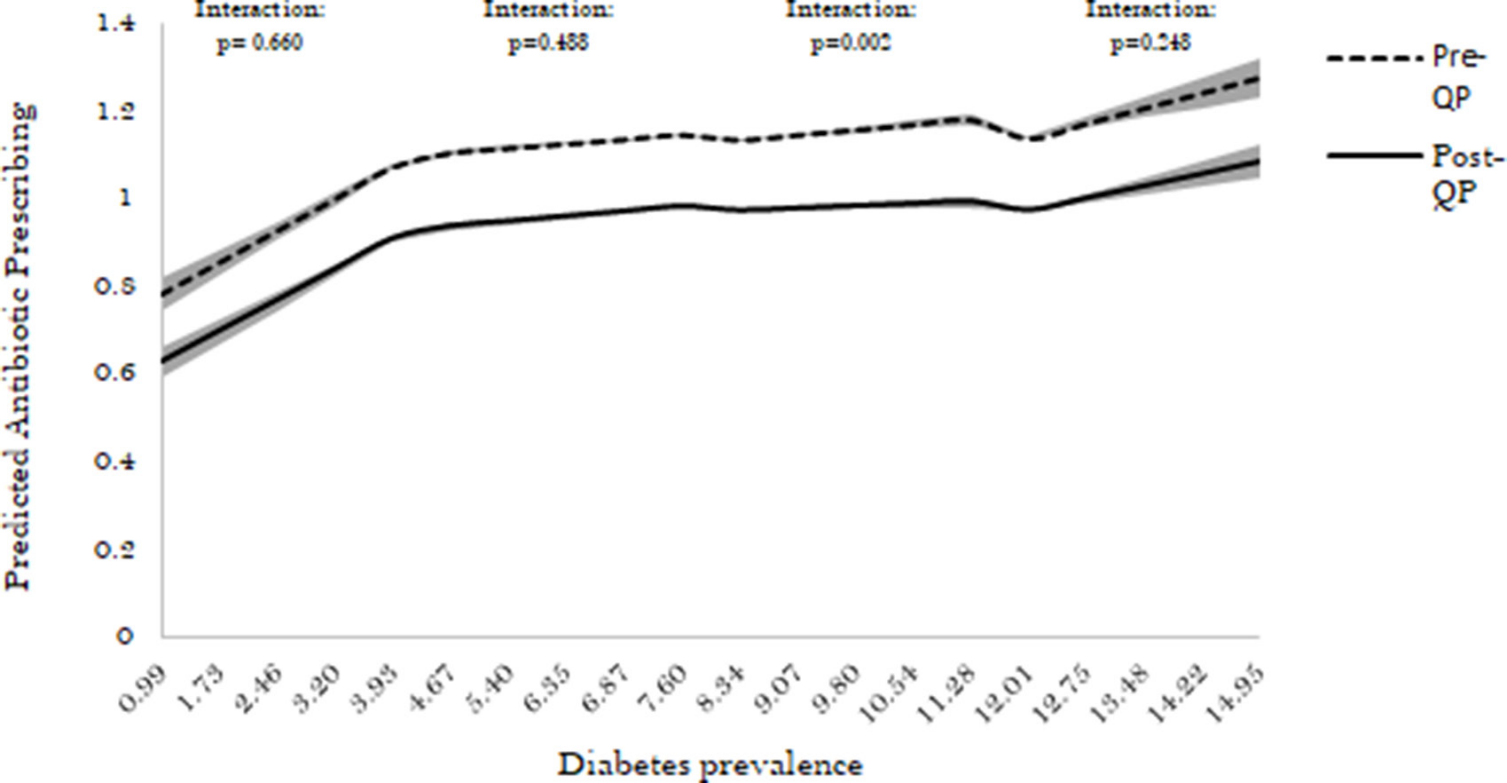
Association between diabetes prevalence and antibiotic prescribing before and after the 2015–2016 QP (for diabetes prevalence spline terms). The shaded portion around the line represents the 95% CI. QP = Quality Premium

For the interaction between QP and diabetes prevalence, a significant variation in prescribing behaviour before versus after QP was seen only in practices with diabetes prevalence of between 7.60% and 11.28% (Figure 2). Before the QP, a 1% higher diabetes prevalence in these practices was associated with +0.016 items per STAR-PU greater prescribing (95% CI = +0.012 to +0.021). This changed to +0.007 items per STAR-PU (95% CI = +0.002 to +0.011) per 1% higher diabetes prevalence after the QP (interaction *p*<0.001).

**Figure 3.**
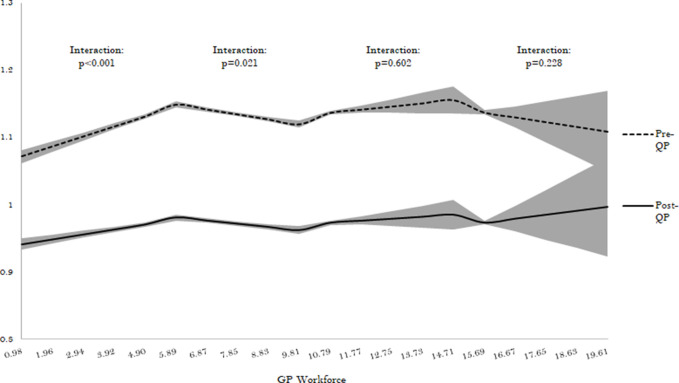
Association between GP workforce and antibiotic prescribing before and after the 2015–2016 QP (for workforce spline terms). The shaded portion around the line represents the 95% CI. QP = Quality Premium

The study also found a significant interaction in practices where GP headcount was fewer than 4.91 and those between 4.91 and 9.81 per 10 000 patients. Following the implementation of QP, the understaffed practices (with fewer than 4.91 GPs per 10 000 patients) experienced a decrease in the pre-QP increasing trend in antibiotic prescribing (difference -0.008 items per STAR-PU per increase; 95% CI = -0.010 to -0.005) (interaction *p*<0.001) (Figure 3). This was different among practices with between 4.91 and 9.81 GPs per 10 000 patients where the pre-QP reduction associated with a higher GP workforce slightly attenuated. The study found no evidence of significant interaction between QP and other spline terms for workforce (interaction *p*>0.23).

The reduction in antibiotic prescribing after the implementation of the QP was similar for all subgroups of practices based on deprivation (interaction *p*>0.13) or respiratory diseases prevalence (interaction *p*>0.29 for all spline terms).

## Discussion

### Summary

Using a longitudinal dataset covering 24 months, the study found that variations in practice characteristics did not strongly affect estimates of the impact of the 2015–2016 QP on antibiotic prescribing in primary care practices in England. The consistency of the immediate and month-on-month effects after accounting for differences in practice characteristics indicates the inclusiveness of the QP in reaching diverse populations. Although consistent in both models, the gradual month-on-month increase after the dip in prescribing at the implementation of the intervention indicates issues of sustainability.

The study also found a differential effect of the 2015–2016 QP on subgroups of practices with a significantly greater reduction seen among high-prescribing practices, understaffed practices, and those with a higher prevalence of comorbidities. The greater reduction among high-prescribing practices might be explained by the targeted implementation of the QP by CCGs on these practices who have more need to reduce prescribing (QP is implemented at CCG level although the outcome is measured at practice level). The reduction reported in practices with higher diabetes prevalence indicates their ability to work towards reducing prescribing rate, while coping with other needs arising from the complexity of their patient population. This finding is important considering the increasing trend in diabetes prevalence in the UK,^29^ and the higher antibiotic prescribing in patients with diabetes owing to higher susceptibility to infection and infection-related adverse outcomes.^25,30,31^

### Strengths and limitations

To the authors’ knowledge, the study is the first to account for other possible explanations of the effect of the QP on antibiotic prescribing in primary care practices (practice workforce size, prevalence of comorbidities, prescribing rate of non-antibiotic drugs, and deprivation), strengthening the evidence on the effectiveness of this financial incentive scheme. A large dataset was used including most (82.8%) practices in England over the observation period, with 12 months observations before as well as after the implementation to capture the temporal trend in antibiotic prescribing.

One of the limitations of the study is that some practice characteristics that could be associated with antibiotic prescribing behaviours, such as consultation rates^32^ and severity of illness, have not been accounted for in the analyses as these data are not available nationally at practice level.

Also, it is recognised that the QP is not the only antibiotic stewardship intervention in England in the period covered by this study;^33^ this limits the causal interpretation of the observed effect of the 2015–2016 QP. However, the QP is the most relevant difference in antibiotic stewardship between 2014–2015 and 2015–2016 financial years, as most of the national interventions, such as the TARGET toolkit (introduced in 2012),^34–36^ and the chief medical officer’s letter to high prescribers (introduced in 2014),^37^ were implemented in both periods.

### Comparison with existing literature

Financial incentives have been used to improve performance and quality of care in different clinical areas and settings.^11,12,38–42^ Evaluation studies have reported mixed effects from such incentive schemes. While the finding on the immediate effect of the 2015–2016 QP on antibiotic prescribing confirms those of other UK studies on this incentive scheme,^11,12^ some studies have shown a limited effect of similar schemes as seen in the month-on-month increase post-QP reported in the study. A study in the Netherlands showed a limited, temporary effect of a one-off behaviour independent financial bonus on the volume of drug prescriptions and the quality of prescribing behaviour in general practice.^40^ Similar results were reported in a UK study that demonstrated reduction in some measures of quality of care following the removal of a financial incentive scheme to improve clinical performance.^43^ However, unlike the QOF evaluated in the study by Minchin *et al.*,^43^ QP is paid at CCG level only (individual GP practices did not receive financial bonuses based on performance), and CCGs need to meet other core criteria to receive the reward.

Furthermore, evaluations of other antibiotic stewardship interventions on high prescribers have also shown mixed effects. A behaviour change intervention that provided social norm feedback to high-prescribing primary care practices in England on their prescribing behaviour showed an overall significant impact in reducing antibiotic prescribing.^37^ However, a similar randomised clinical trial in Switzerland demonstrated that personalised prescription feedback to physicians to reduce antibiotic prescribing in primary care made no significant difference in overall antibiotic prescribing.^44^ These studies have mostly focused on interventions that are specifically designed to target high prescribers alone.

### Implications for research and practice

The study provides novel evidence on the differential effect of the 2015–2016 QP. Highlighting the ability of high-prescribing practices, understaffed practices, and those dealing with a higher prevalence of comorbidities to make substantial improvements when adequately supported; this study emphasises the role of CCGs in identifying and working with these practices.

The substantial scope for change in high-antibiotic prescribing practices illustrated by this study should prompt these practices to take action on this issue, and to actively seek support from CCGs in addressing overprescribing. Also, policymakers and antibiotic stewardship programmes should target these practices specifically in the design and targeted implementation of antibiotic stewardship interventions. The shortage of GPs in NHS primary care is of increasing concern, and the greater effect on understaffed practices underlines the need for appropriate resourcing. The current government’s promises regarding increased GP numbers notwithstanding,^45^ CCGs have an important responsibility to identify and work with understaffed practices in the implementation of antimicrobial stewardship interventions to facilitate performance improvements.

The gradual increase in antibiotic prescribing in the months following the 2015–2016 QP (countering some of the immediate effects of the intervention) demonstrates the need for more efforts towards the sustainability of the effects of AMS interventions. Approaches to maintain or improve the immediate effects of such interventions should be considered at the design stage.
